# Detection of low-frequency resistance-mediating SNPs in next-generation sequencing data of *Mycobacterium tuberculosis* complex strains with binoSNP

**DOI:** 10.1038/s41598-020-64708-8

**Published:** 2020-05-12

**Authors:** Viola Dreyer, Christian Utpatel, Thomas A. Kohl, Ivan Barilar, Matthias I. Gröschel, Silke Feuerriegel, Stefan Niemann

**Affiliations:** 1 Molecular and Experimental Mycobacteriology, Research Center Borstel, Borstel, Germany; 2 German Center for Infection Research, Partner Site Hamburg-Lübeck-Borstel-Riems, Borstel, Germany

**Keywords:** Microbiology, Molecular biology

## Abstract

Accurate drug resistance detection is key for guiding effective tuberculosis treatment. While genotypic resistance can be rapidly detected by molecular methods, their application is challenged by mixed mycobacterial populations comprising both susceptible and resistant cells (heteroresistance). For this, next-generation sequencing (NGS) based approaches promise the determination of variants even at low frequencies. However, accurate methods for a valid detection of low-frequency variants in NGS data are currently lacking. To tackle this problem, we developed the variant detection tool binoSNP which allows the determination of low-frequency single nucleotide polymorphisms (SNPs) in NGS datasets from *Mycobacterium tuberculosis* complex (MTBC) strains. By taking a reference-mapped file as input, binoSNP evaluates each genomic position of interest using a binomial test procedure. binoSNP was validated using *in-silico, in-vitro*, and serial patient isolates datasets comprising varying genomic coverage depths (100-500×) and SNP allele frequencies (1-30%). Overall, the detection limit for low-frequency SNPs depends on the combination of coverage depth and allele frequency of the resistance-associated mutation. binoSNP allows for valid detection of resistance associated SNPs at a 1% frequency with a coverage ≥400×. In conclusion, binoSNP provides a valid approach to detect low-frequency resistance-mediating SNPs in NGS data from clinical MTBC strains. It can be implemented in automated, end-user friendly analysis tools for NGS data and is a step forward towards individualized TB therapy.

## Introduction

Globally, tuberculosis (TB) is the leading cause of death from a single infectious agent with an estimated 1.3 million deaths and 10 million new TB cases in 2017^1^. The emergence of drug-resistance challenges global TB control efforts with 558 000 estimated cases in 2017 being resistant to the frontline drug rifampicin (RMP); 82% of those were classified as multidrug resistant (MDR) strains, defined as showing additional resistance against isoniazid (INH)^1^ and even 10% of those were estimated to be extremely drug resistant (XDR) which means carrying further resistances to a quinolone and one injectable drug^1^. Early case detection, rapid drug susceptibility testing (DST), and effective treatment are core elements of global TB programs to control the spread, emergence, and transmission of resistant strains^2^.

Resistance of *Mycobacterium tuberculosis* complex (MTBC) strains is caused by spontaneous mutations, mainly single nucleotide polymorphisms (SNPs), in specific regions of the pathogen’s genome. In general, mutations appear by chance with a probability of between 10^-6^ and 10^-8^ per generation depending on the observed locus^3^. Normally, mutations in resistance associated genes are associated with a fitness cost, however, under a selection pressure such as antibiotic treatment resistant cells are selected and fixed in the population^4^.

The current gold standard to determine drug resistance in clinical MTBC strains is broth-based phenotypic drug susceptibility testing DST (pDST). While pDST can be carried out on solid or liquid medium, all culture-based testing methods are limited by the slow growth of the pathogen and require at least eight to twelve weeks (solid medium) or seven days to six weeks (liquid medium) before results are available^5–7^. Additionally, pDST yields poorly reproducible results for certain drugs such as pyrazinamide (PZA), streptomycin (SM) and ethambutol (EMB)^8–10^. Alternatively, PCR based molecular tests based on processed patients’ specimens such as line probe assays are faster compared to phenotypic tests and allow the detection of resistance markers for a limited number of drugs^11–13^. However, their analytical capacity is restricted by the test format, e.g. the small number of interrogated mutations^11–13^. Instead, whole-genome sequencing (WGS) using next-generation sequencing (NGS) technologies enables a more comprehensive analysis of genomic resistance-associated variants^14^. Several studies showed good performance for genotypic resistance prediction using NGS, especially for the most important first line drugs INH and RMP with a sensitivity and specificity of 0.975 (95% CI 0.952 - 0.989) and 0.996 (95% CI 0.993 - 0.99.8) for INH and 1 (95% CI 0.971 -1) and 0.992 (95% CI 0.989 – 0.995) for RMP, respectively^15–20^. Moreover, by using NGS for resistance prediction, the turnaround time to obtain results can be shortened to 5 days starting from a primary culture^21,22^.

Both, genotypic and phenotypic DST are challenged by heteroresistant strain populations comprising both susceptible and resistant bacterial cells in parallel^23–25^. Heteroresistance can emerge during treatment when a small subpopulation of e.g. 5% of bacterial cells carries the mutation that confers resistance to the drug used^24–26^. As a consequence, the duration to obtain the complete resistance profile of the infecting strain by pDST can be prolonged up to 42 days^5^.

Previous studies suggest that genotypic methods are able to detect heteroresistance in MTBC samples, but the detection limit of pDST (1%) is far from being reached^26,27^. Directly connected to this, Folkvardsen *et al*. demonstrated in a recent comparison of pDST with genotypic DST for populations comprising varying proportions of resistant bacteria, that the lowest frequency (1%) of resistant cells could only be detected by means of pDST^23,26^. Another study investigated the performance of commonly used methods for the detection of low-frequency variants in targeted sequencing experiments noted that valid variant callers for low-frequency variant detection require a special library preparation such as the target enrichment via DNA hybridization capture^27^.

Missing resistant subpopulations by culture- and genome-based resistance diagnostics, however, is leading to erroneous resistance profiles, inefficient treatment regimens, and consequently, to treatment failure, resistance development and further spread of resistant bacteria^28^. Accordingly, the detection of low-frequency resistance-mediating variants is crucial for accurate molecular resistance prediction as basis for effective treatment regimens^28^.

NGS-based genome analysis has the potential power to overcome this challenge if sufficient read depth is achieved. However, the majority of NGS bioinformatics data analysis workflows are not tailored to or even lack the ability to detect minority alleles at heterogeneous sites in the genome of MTBC strains.

Considering the increased capacity to use genomic sequence data in the diagnosis and creation of personalized treatment regimens for TB patients, we developed and evaluated binoSNP, a variant detector especially designed to detect low-frequency SNPs in MTBC strains based on a statistical approach. Our special focus in this study was to investigate, how coverage and minimal detectable allele frequency are related and with which coverage it is possible to reach the 1% detection threshold of pDST.

We validate the ability of binoSNP to detect low-frequency SNPs at varying coverage depths and allele frequencies with NGS datasets from *in-silico, in-vitro* and clinical samples.

## Results

### The binoSNP tool

**Figure 1:**
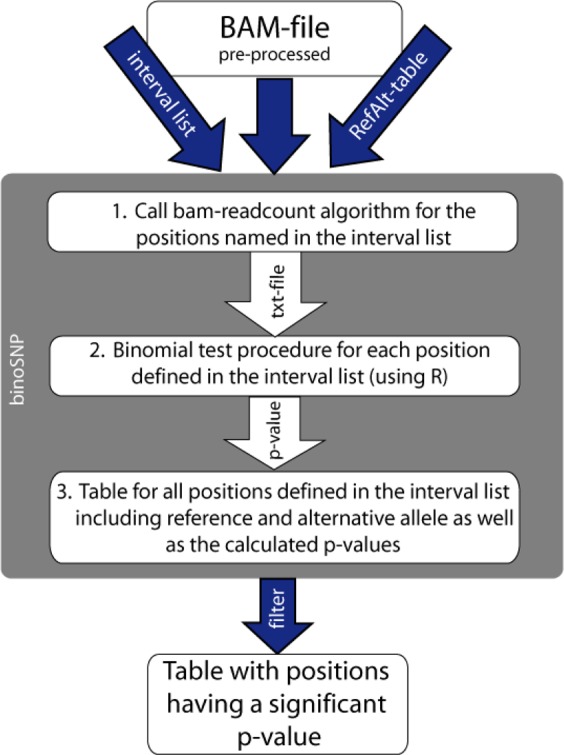
Schematic overview of the binoSNP workflow. binoSNP accepts a preprocessed BAM-file where ideally duplicates (PCR artefacts) have been removed and base quality scores have been recalibrated. Additionally, the script requires an interval list where the positions to be examined are named as well as a RefAlt-table defining reference and the alternative allele for those positions. As a first step the bam-readcount algorithm from Larson^29^ is executed to extract information about the number and quality of reference and alternative alleles at the positions named in the interval list and stores this information in a text file. In a second step the resulting txt-file is read into R and for each position a p-value is calculated by using the binomial test procedure. In the next step a table is produced containing all information including the calculated p-value for each position named in the interval list. The last step applies the user-defined p-value, e.g. report variants with a p-value below 5% (standard value for statistical significance)

To optimize the detection of low-frequency SNPs in NGS data of clinical MTBC isolates, we developed the binoSNP tool. binoSNP is written in perl integrating functionality of R and the program bam-readcount^29^, and is available on GitHub (www.github.de/ngs-fzb/binoSNP). A schematic overview of the established workflow is shown in Fig. 1. Although developed for the detection of low-frequency associated SNPs in MTBC, this method can be applied to other bacterial pathogens and is not limited to the known resistance positions of MTBC.

This tool employs a statistical algorithm to infer whether each of the genomic positions analyzed are heterogeneous. Taking reference-mapped NGS data in the BAM format as input, binoSNP analyzes a user-defined list of positions, with a set of known resistance-associated positions being used as default (Supplementary Table S1). For each position, the tool calculates a p-value based on a binomial test describing the probability that the observed number of non-reference alleles (alternative alleles) is due to sequencing errors. This test assumes that both sequencing errors over the aligned reads are equally distributed and that reads are independent from each other.

The main input for the tool are reference-mapped NGS data from MTBC or other bacterial pathogens in the BAM format. Although each standard BAM-file could be used as input, it is recommended to process the files as described in the methods section “NGS pipeline”. Additional input comprises a list of positions to be investigated for low-frequency mutations (interval list) and the corresponding table defining reference and alternate allele for those positions (RefAlt-table). The tool includes predefined position lists and RefAlt-tables for resistance positions of TB (Supplementary Table S1), which can be used as example files and can be edited by the user.

### Coverage simulation

**Figure 2:**
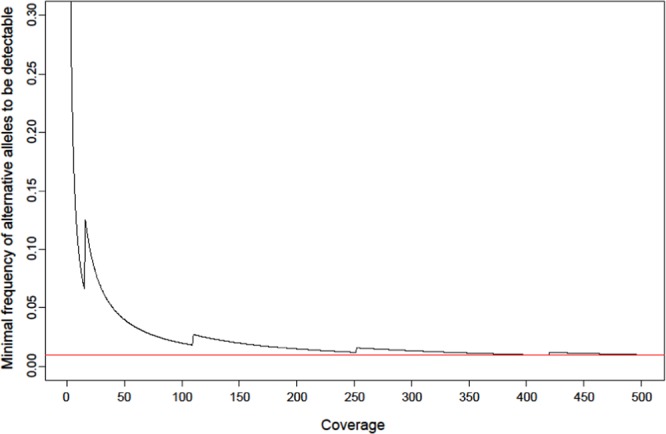
Minimal detectable allele frequency. The coverage is displayed on the x-axis and the minimum frequency of alternative alleles which is needed to get a significant result (here p-value < 0.05) is shown on the y-axis. For the simulation an error probability of 0.00326 (base quality score Q25, which can be transformed to an error probability of 0.00316 + Illumina sequencing error of 0.01%) has been assumed. Calculation was done with R. The red line illustrates that with a coverage of 400× the minimal detectable frequency of alternative alleles is 1%.

To get an estimation of the minimum number of reads required to get statistically significant results for low-frequency SNP detection using the described binomial test procedure, we first conducted a computer simulation of varying sequence read coverages between 1x and 500×(Fig. 2). The binomial test procedure was performed with a success rate, in this case more precisely sequencing error probability, of 0.00326, meaning that we assumed a mean base quality score of Q25 plus the Illumina error rate of 0.01%. For the simulation, a 0.05 p-value was considered statistically significant. The minimum detectable frequency is calculated by dividing the minimum number of alternative bases leading to a p-value < 0.05 by the respective coverage. This analysis illustrates that the higher the coverage, the lower the minimum detectable frequency of alternative bases is (Fig. 2). Our simulation data suggest that a non-reference allele at 5% frequency (n = 3) can be validly detected with a coverage of approx. 50×, and a non-reference allele at 1% frequency (n = 4) with a coverage of at least 400× (red horizontal line Fig. 2).

### *In-silico* validation

**Figure 3:**
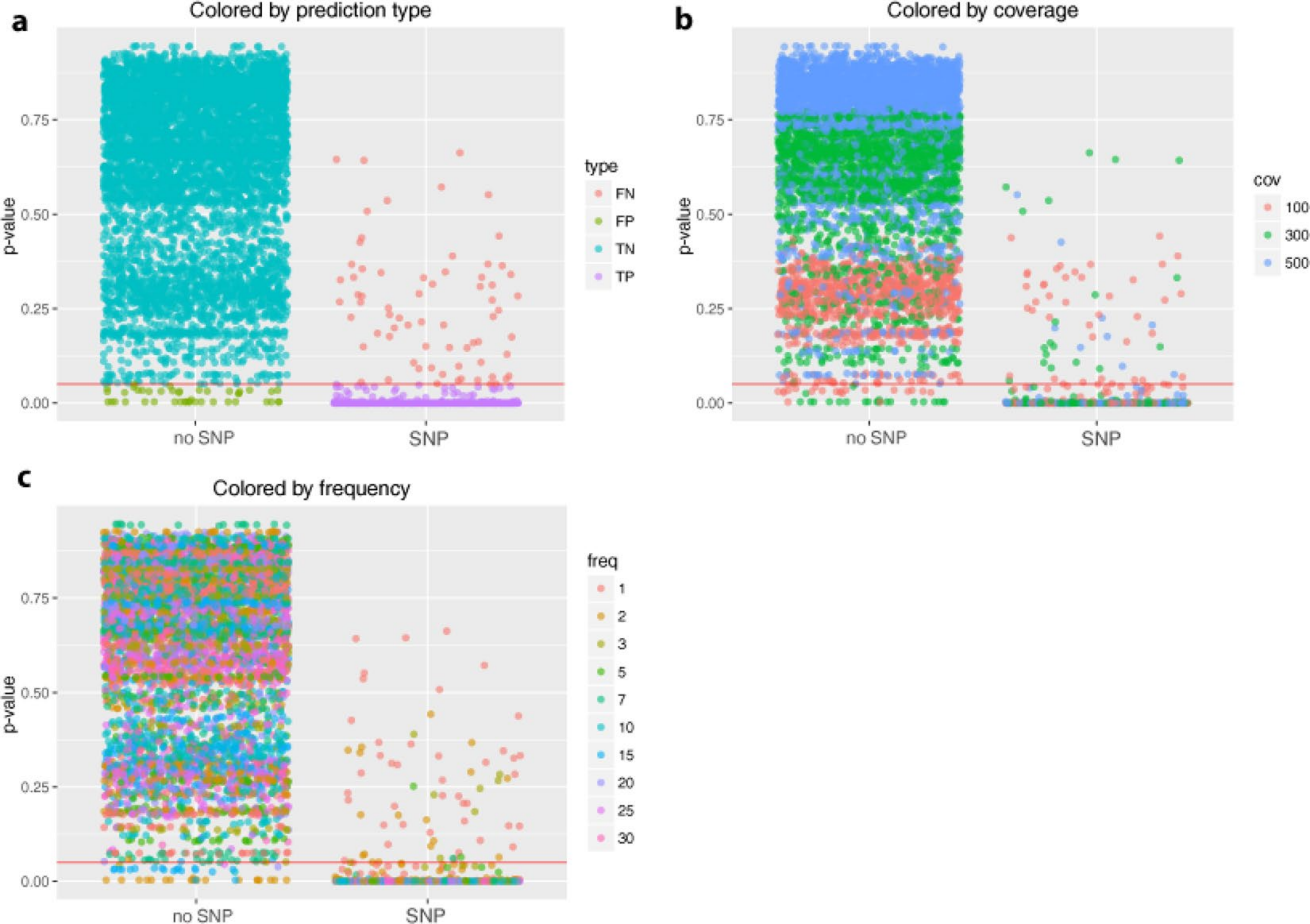
p-value distribution of *in-silico* dataset positions. Scatterplot of calculated p-values for all positions with at least one alternative allele (n = 6870) divided by the status no SNP (n = 5955)/SNP (n = 915). The red line marks the critical p-value of 5%. (**a**) SNPs colored by the prediction type. FN – false negative, FP – false positive, TN – true negative and TP – true positive. The majority of positions was correctly classified, only 44 positions were false positive and 69 were false negative. (**b**) Colored by the theoretical coverage observed at a position; 56% of the wrongly classified positions had 100x coverage. (**c**) Colored by the theoretical frequency of the alternative allele at a position. Only positions with less than 5% frequency were wrongly classified.

Next, the binoSNP algorithm was evaluated using 600 *in-silico* generated FastQ datasets containing various resistance-mediating variants in different genes along with different coverage-allele frequency combinations (Table 1). For the evaluation of binoSNP results, we included all positions of Table 1 if at least one alternative allele was present in the *in-silico* validation dataset. In total, a set of n = 6870 judged (p-value) positions of which 915 (13%) are SNPs and the others are wildtype were analyzed. Using a threshold $\,{p}_{s}$ = 0.05 (red line), 5911 (99.3%) positions were true negative, meaning no SNP was predicted (Fig. 3A). binoSNP correctly detected 864 SNPs (92.5%), but missed 69 SNP positions that should have SNP calls based on the construction of the data. However, inspection of these positions revealed that the majority (n = 53, 76.8%) indeed showed only 1–2 alternative alleles and none of these had a frequency higher than 2.5% alternative allele (Fig. 3C, Supplementary Table S4). For 44 positions binoSNP wrongly detected a SNP at a “wildtype” position, for which the majority (n = 24, 54.5%) had at least 3 alternative alleles, pointing to an error in the simulation algorithm and not the SNP detection (Supplementary Table S4).

**Table 1:** *In-silico* datasets and included resistance-mediating mutations.

Dataset	Mutation
MDR1	*rpoB* His445Asn
	*katG* Ser315Asn
MDR2	*rpoB* His445Arg
	*katG* Trp300Cys
MDR3	*rpoB* Ser450Leu
	*fabG1* -8 T/A
XDR1	*rpoB* His445Asn
	*katG* Ser315Asn
	*rrs* 1401 A/G
	*gyrA* Asp94Asn
XDR2	*rpoB* His445Arg
	*katG* Trp300Cys
	*rrs* 1401 A/G
	*gyrA* Asp94Ala
XDR3	*rpoB* Ser450Leu
	*fabG1* -8 T/A
	*rrs* 1484 G/T
	*gyrB* Thr500Asn
PZA1	*pncA* Val163Ala
PZA2	*pncA* His82Asp
EMB1	*embB* Met306Leu
EMB2	*embB* Gly406Ser
EMB3	*embB* Gly406Ala
SM1	*rpsL* Lys43Arg
SM2	rpsL Lys88Arg
RMP1	*rpoB* Asp435Val
RMP2	*rpoB* Ile491Phe
INH1	*katG* Ser315Gly
INH2	*fabG1* -15 C/T
FQ1	*gyrB* Asp461Asn
FQ2	*gyrB* Glu501Asp
FQ3	*gyrA* Ser91Pro

Overall, binoSNP showed a sensitivity of 0.92 [0.91, 0.94] and a specificity of 0.99 [0.99, 0.99] to detect a heterogeneous SNP with the coverage-allele frequency combinations tested (Supplementary Table S2). Sensitivity improves with higher coverage or the restriction to higher minimal frequencies. For example, repeating the analysis with coverages of at least 100x and SNPs with a frequency of >3% the sensitivity to detect the resistance associated SNPs is 0.99 [0.99, 1.00] (Supplementary Table S2).

**Figure 4:**
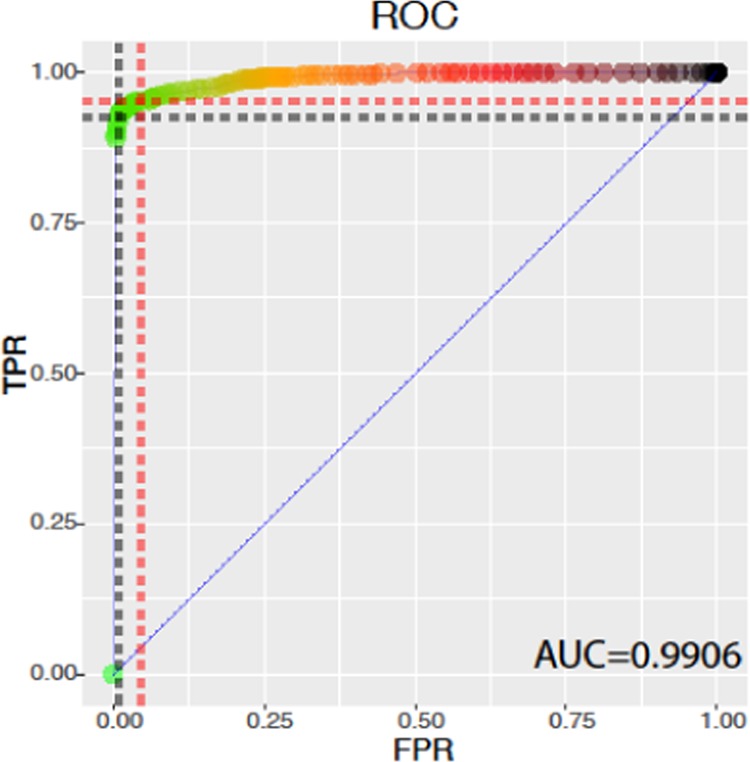
Performance measurement of separating SNPs and artefacts using p-value by. a Receiver Operating Characteristics (ROC) curve. ROC curve based on p-values with false positive rate (FPR) on the x-axis and true positive rate (TPR) on the y-axis. The area under the curve (AUC) equals 0.9906 (99.06%), which means it has near optimal measure of separability. The black dashed line marks the cut-offs for $\,{p}_{s}=0.05$. The theoretical optimal threshold for a p-value to distinguish between true SNPs and sequencing errors based on the ROC curve was calculated as ${p}_{g}=0.18$ and is shown as red dashed line

To measure how well the calculated p-value can distinguish between SNPs and sequencing errors we calculated a Receiver Operating Characteristics (ROC) curve (Fig. 4). The area under the curve (AUC) of the ROC curve, which is interpreted as a quality value of the ROC curve based on p-values, is 0.9906 (99.06%), which means it has near optimal measure of separability (Fig. 4). Here, 915 SNP positions and 5980 non-SNP positions were included. The theoretical optimal threshold for a p-value to distinguish between true SNPs and sequencing errors based on this ROC curve was calculated as ${p}_{g}=0.18$ (Fig. 4, red dashed line), opposed to the used ${p}_{s}=0.05$ for the evaluation of simulation data. Using ${p}_{g}$ as separator there were only 43 false negative SNPs, but the number of false positives increased from 69 with ${p}_{s}$ to 256 with $\,{p}_{g}$. That translates to a higher sensitivity 0.95 [0.94, 0.97] when using ${p}_{g}$, but simultaneously, the specificity decreased to 0.96 [0.95, 0.96] (Supplementary Table S2). These results show, that a stricter p-value e.g. of 0.05 leads to more specific results along with high sensitivity.

### *In-vitro* validation

As the evaluation with *in-silico* datasets showed convincing results, we next produced *in-*vitro datasets by mixing DNAs from *M. tuberculosis* H37Rv strains carrying single mutations in *rpoB* (RpoB Ser531Leu and RpoB His526Pro) and wildtype parental *M. tuberculosis* H37Rv reference strain (ATCC 27294) at different ratios (1:99, 5:95 and 10:90) of mutant/wildtype DNA (see Methods). NGS data were obtained with an average coverage of 600× and processed as described in the methods section followed by variant calling with binoSNP. As shown in Table 2, only the correct RpoB mutations Ser531Leu and His526Pro received a p-value <0.05 for each dataset, illustrating the robustness of our approach. Notably, the mixtures with 1% resistant subpopulations were correctly detected with a statistically significant p-value. Calling variants with binoSNP in the *in-vitro* datasets resulted in a sensitivity and specificity of 100%. Furthermore, coverage of 394×(Table 2) was sufficient for yielding a correct call for a SNP with 1% allele frequency, which is consistent with the coverage simulation.

**Table 2:** Analysis results for *in-vitro* datasets.

Mixture	Position	REF	ALT	DP	Freq (ALT)	Q (ALT)	p-value	AA exchange
RpoB526_1	761140	A	C	394	0.0076	36.67	5.99 × 10^−04^	RpoB His526Pro
RpoB526_5	761140	A	C	443	0.0542	35.42	5.67 × 10^−35^	RpoB His526Pro
RpoB526_10	761140	A	C	586	0.0819	35.58	1.14 × 10^−82^	RpoBHis526Pro
RpoB531_1	761155	C	T	492	0.0203	32.80	2.1 × 10^−06^	RpoB Ser531Leu
RpoB531_5	761155	C	T	656	0.0503	31.79	4.52 × 10^−53^	RpoB Ser531Leu
RpoB531_10	761155	C	T	582	0.1031	31.52	3.27 × 10^−118^	RpoB Ser531Leu

Abb.: REF - Reference allele, ALT – Alternative allele, DP – Coverage/read depth, Freq – Frequency, Q – Base quality value, AA – Amino acid.

### Validation in a clinical setting

Finally, to assess the potential application of binoSNP in clinical practice, it was used to analyze NGS datasets from clinical MTBC isolates from a published study conducted in Uzbekistan between 2003 and 2008^30^. The NGS data were obtained from serial isolates of patients, who developed ofloxacin (OFX) resistance during treatment while being infected with the same strain over time shown by IS*6110* DNA fingerprint at that time. By applying binoSNP we were able to detect heterogeneous positions in resistance associated genes in 23 out of 53 serial isolates (Supplementary Table S3). Overall, for ten patients at least one resistance-mediating SNP in one serial isolate was detected that was present in less than 75% of the reads at a position (Supplementary Table S3). The lowest detected frequency found in this dataset was 5% alternative allele. These SNPs would have not been detected by applying standard NGS diagnostic thresholds (e.g. >75%)^31^, underlining the importance of dedicated low-frequency analysis workflows in NGS-based resistance prediction.

## Discussion

In this study, we present the possibility to detect already small subpopulations of resistant MTBC strains, by calling low-frequency SNPs, involved in resistance development of clinical MTBC strains. The established method, called binoSNP, showed an excellent performance for the detection of heterogeneous resistance variants in *in-silico*, *in-vitro* and clinical data sets. We showed that the detection limit for low-frequency resistance variants strongly depends on the read coverage. The higher the coverage, the lower is the possible detectable frequency. For *in-silico* datasets with 1, 3, 5, 7, 10, 20 and 30% alternative allele and coverages of 100, 300 and 500×, respectively, binoSNP showed an overall sensitivity of 0.92 [0.91, 0.94] and a specificity of 0.99 [0.99, 0.99] to detect a resistance associated SNP. To increase the sensitivity for detecting the respective resistance SNP in the *in-silico* samples, coverages should be restricted to a value of at least 100× and a frequency of >3% (Supplementary Fig. S1).

A theoretical calculation of the minimal detectable number of alternative alleles depending on different coverages from 1 to 500× suggested a threshold of at least 400× coverage to detect 1% of the alternative allele. These data show superior results compared to an experimental study from Spencer *et al*.^27^, who suggested a detection threshold of 2% minority allele with at least 500× coverage. Our tests with *in-silico* NGS datasets (validation datasets) with 300–500× coverage indicate that binoSNP accurately calls 1% resistant subpopulations with high accuracy, which is the critical value to be comparable with pDST. Indeed, by analyzing NGS datasets from DNA mixtures of *in-vitro* selection clones with their parental strains, we showed that binoSNP is able to detect low-frequency resistance variants at 1% level with 394× coverage.

Predicting the resistance phenotype from genome sequences has a number of advantages compared to culture-based DST. It allows for the detection of resistance variants for virtually all resistances including drugs for which pDST has a low performance or may not yet be available^14^. Indeed, we could recently show that genomic resistance prediction for first line drugs has reached a precision sufficient for clinical use and can, thus, replace pDST for first line drugs^20^. While the overall accuracy of NGS-based resistance prediction has reached a tremendous level, several challenges e.g. easy data interpretation and the detection of low-frequency variants remain to be solved^14,20,32^.

The low-frequency problem can potentially be overcome by automated tools providing a workflow for detection and statistical evaluation of minority variant populations. With binoSNP, we reached a similar detection threshold for minority variants than obtained by pDST (1% of resistant subpopulations) using a semi-automated NGS data analysis workflow. Compared to pDST, overall time to result can be much faster, especially in case of small subpopulations, where pDST is very time-consuming with processing times of up to 42 days^5,33^. This potentially leads to suboptimal treatment regimens that foster the spread of resistant bacteria as well as the development of additional resistance-mediating mutations. In contrast, processing time of direct NGS from sputum samples can be shortened to 5–7 days ending up with a complete resistance profile^21,22,34^. Although binoSNP has been evaluated with NGS data generated from cultures, the error term can easily be adjusted for different techniques such as sequencing from sputum samples or targeted NGS sequencing.

Molecular detection tools such as the GeneXpert or Hain line probe assay are able to detect resistance mutations to particular drugs within hours directly from clinical samples. However, resistance detection is limited to specific mutations and the detection of small subpopulations (less than 10%) is not possible^23,26^. A study from Zetola *et al*. showed that the GeneXpert was unable to detect resistance *in-vitro* as long as less than 90% of the population harbor the respective mutation^35^.

The results obtained here, show a much better performance of NGS to detect low-frequency variants even at 5% to 1% level. However, valid detection of 1% minority populations still requires a high coverage of 400×, which is currently not often targeted in routine diagnostic workflows. Realistic values range between 50× and 150× coverage per sample. Using binoSNP and a threshold of p < 0.05 in a dataset with 100x coverage, we observed a sensitivity of 0.86 [0.82, 0.90] and specificity of 0.98 [0.96, 0.98] including all tested allele frequencies. The sensitivity can be increased to 0.99 [0.96, 1.00] by restricting the allele frequency to >3% (Supplementary Figure S3). However, sensitivity and specificity, including also the smaller frequencies, improve with increasing coverage (Supplementary Figure S4).

Still, the specific role of low-frequency variants during treatment failure is not completely understood, and only few papers confirm the clinical importance^36–38^. This is partially due to the lack of valid detection methods. However, recent papers indicate that low-frequency variants appear in clinical samples and are related to treatment failure and resistance development^26,35,39,40^. With binoSNP, we developed a tool allowing for the accurate detection of low-frequency variants in NGS datasets from clinical MTBC strains, thus providing in depth insights into the development of the resistome with a detailed view on subpopulations carrying particular resistance mutations. Indeed, using binoSNP, we showed that resistance development in serial isolates from 13 patients from Uzbekistan was more complex than previously assumed^30^. Low-frequency (<75%) resistance-mediating SNPs occurred in isolates from ten patients, which were not detected applying standard NGS data analysis procedures, normally applying SNP calling thresholds of more than 75%^31^.

binoSNP is based on a statistical analysis and results are dependent on the p-value the user chooses as significant. While our *in-silico* data analysis suggested a ${p}_{g}=0.18$ as the optimal p-value for the detection threshold in the applied methodological framework, we would suggest a p-value of 0.05 for the detection of low-frequency variants to maximize specificity with a low cost of sensitivity. Indeed, applying a p-value below 0.05 for filtering in our simulated dataset resulted in high sensitivity and specificity. In addition, the majority of “false positive” and “false negative” SNP calls result from the construction of the NGS data simulation algorithm that not exactly produced low-frequency SNP values at the expected positions.

Overall, the ROC curve suggests that the p-value calculated by the binomial test procedure is an accurate separator to distinguish between actual variants and artefacts. The standard variant calling tools from SAMtools and GATK do not reach a similar detection threshold as shown by Spencer *et al*.^27^. In that study the authors compared, among others, the performance of the variant callers from SAMtools and GATK to detect SNP subpopulations in targeted NGS data with a depth of >1000 reads. SAMtools performed inferior to GATK with a mixed base call detection rate of only 49% and a frequency of at least 25%, while GATK’s algorithm showed better performance with a detection rate of 97% for mixed base calls with a frequency of at least 20%. Below this allele frequency threshold, the performance decreased dramatically with a sensitivity of 0% for SAMtools detecting SNPs with 20% alternative allele frequency and a sensitivity of 21% for GATK detecting SNPs with 10% alternative allele frequency^27^.

In conclusion, binoSNP is a new approach to detect and statistically evaluate SNPs including low-frequency variants in resistance genes from NGS data of MTBC strains. binoSNP showed a high sensitivity and specificity for detection of low-frequency SNPS even at 1% level, and provides a statistical evaluation of the SNP calls. binoSNP will definitely foster the integration of NGS-based resistance predictions into daily diagnostics, thus, improving the timely detection of resistance patterns and enabling precision treatment of MDR/XDR TB patients.

## Methods

### NGS pipeline

All FastQ-files were processed via a reference-based approach. In a first step all reads were mapped to the reference sequence *M. tuberculosis* H37Rv (GenBank accession number NC_000962.3) using BWA-MEM^41^. The initial mapping was improved by further processing of the BAM-file. For this task duplicates were removed using SAMtools^42^ and base quality score recalibration and realignment around small insertions or deletions (indels, 1-30 bp) was performed using the tools BaseRecalibrator, RealignerTargetCreator and IndelRealigner from the Genome Analysis Toolkit (GATK) Version 3^43,44^.

### *In-vitro* sample preparation

For setting up *in-vitro* test datasets the DNA of the reference lab strain *M. tuberculosis* H37Rv ATCC 27294 was spiked with DNA of two different mutated *M. tuberculosis* H37Rv strains carrying the two most frequent RMP resistance mutations RpoB Ser531Leu (clone SR1a) and His526Pro (clone SR4k). The mixtures contained 1%, 5% and 10% of the respective mutant strain. Library preparation for the DNA-mixtures was carried out with the Illumina Nextera XT preparation kit and sequenced on the Illumina NextSeq 500 system (151 bp, paired-end) following Illumina’s instructions. Each mixture was sequenced with an average depth of approximately 600× coverage. All samples were analyzed using the described NGS pipeline and binoSNP.

### *In-silico* sample preparation

For assembling *in-silico* test datasets FASTA-files were produced containing the reference sequence *M. tuberculosis* H37Rv (GenBank accession number NC_000962.3) with different resistance-mediating mutations (Table 1) using the tool FastaAlternateReferenceMaker from GATK^44^. As a next step the artificial alternative sequences and the standard reference sequence *M. tuberculosis* H37Rv (GenBank accession number NC_000962. 3) were transformed into FastQ-files using the algorithm dwgsim from Nils Holmer^45^. Subsequently, different proportions of reference FastQ-files and alternative reference FastQ-files were merged to achieve coverages of 100, 300 and 500× and mutation frequencies of 1, 2, 3, 5, 7, 10, 15, 20, 25 and 30% for each dataset shown in Table 1. Overall, 600 paired-end *in-silico* FastQ-files were generated, which were analyzed with the described NGS pipeline and binoSNP.

### Clinical setting

To validate the method within a clinical setting we analyzed data from a study conducted in Uzbekistan between 2003 and 2008^30,46^, which was approved by the Médecins Sans Frontières international ethics review board. This already published study addressed the development of OFX resistance and the development of XDR-TB during MDR-TB treatment^30,46^. At that time the IS*6110* DNA fingerprint method was used to check samples for re- and mixed infection^30^. Of the 87 TB-patients enrolled in that study, 18 isolated TB strains developed resistance to OFX during treatment^30^. In four of these patients, an additional strain was identified in the follow-up samples, one patient had a mixed infection and 13 patients were infected with the same strain according to IS*6110* DNA fingerprint method^30^. For the here presented study we generated NGS data of the TB strains which were isolated from serial sputum samples of these 13 patients using the Nextera XT Library preparation kit and sequenced on the Illumina NextSeq 500 system (151 bp, paired-end) following Illumina’s instructions. The data were analyzed using the described NGS pipeline followed by binoSNP.

### Binomial test procedure

binoSNP includes a statistical rating of heterogeneous positions. This judgement was carried out using the binomial test distribution provided by R. A prerequisite is that sequencing errors are equally distributed over the aligned reads at a specific position and the reads are independent from one another. The random variable describes the number of sequencing errors so that a small p-value represents the probability that the observed number of non-reference alleles at a specific position are sequencing errors appearing by chance given a fixed error probability (${p}_{0}$).

The calculation of the p-value is done using the formula:

$$p={P}_{{p}_{0}}(X\ge k)=\mathop{\sum }\limits_{i=k}^{n}(\begin{array}{c}n\\ i\end{array}){{p}_{0}}^{i}{(1-{p}_{0})}^{n-i}$$

where p_0_ is the probability for a sequencing error calculated as position mean by transformation of base quality score *Q* at a specific position. Transformation was done by the formula:

$$P(error)={10}^{-Q/10}$$

and adding the general error term of Illumina sequencing reads (0.01%), *k* is the number of observed mismatches (alternative alleles) compared to the reference sequence and *n* is the coverage at the respective position (sample size).

### Coverage simulation

For the coverage simulation we have chosen a mean base quality value of Q25, which corresponds with an error probability of 0.00316 (0.3%), which is a medium good value for base quality scores and added the Illumina error rate of 0.01%:

$${p}_{E}=0.00316+0.001=0.00326.$$

The simulation was performed using R statistics version 3.0.1.

### Implementation of binoSNP

binoSNP is implemented as perl script with R integration, as well as the usage of an algorithm called bam-readcount^29^ and is available on GitHub (www.github.de/ngs-fzb/binoSNP).

The analysis workflow of binoSNP is as follows (Fig. 1). At first, the script uses the input BAM-file and calls the bam-readcount algorithm for the defined positions and stores this information into a text file. The text file contains information about the number and base quality of the bases A, C, G and T at a specific position. The next step is the call of a script starting the binomial test procedure using the information from the bam-readcount algorithm which calculates the p-values and stores them in a second table. The last step applies the user-defined filtering, e.g. report variants with a p-value <0.05 (standard value for statistical significance).

## Supplementary information

Supplementary Figures

Supplementary Tables
