# Supplementary material for: Detection of low-frequency resistance-mediating SNPs in next-generation sequencing data of Mycobacterium tuberculosis complex strains with binoSNP
Source: Sci Rep. 2020 May 12;10:7874. doi: 10.1038/s41598-020-64708-8 (PMC7217866; doi:10.1038/s41598-020-64708-8)
Supplement: Supplementary file 1 — Supplementary Figures [file 41598_2020_64708_MOESM1_ESM.docx]

**Supplementary Figures**

**Supplementary Figure S1.** Shown is the proportion of correctly positive positions (n = 846) per coverage for a p-value of 0.05, each subdivided into the theoretically inserted frequency of the alternative allele.

**Supplementary Figure S2.** Shown is the frequency of each prediction type (true positive, false negative, true negative and false positive) per coverage with a p-value of 0.05.

**Supplementary Figure S3.** Shown is the number of alternative alleles of the 69 false negative SNPs. Bars are colored regarding the coverage.

**Supplementary Figure S4.** Shown is the number of alternative alleles of the 44 false positive SNPs. Bars are colored regarding the coverage.****
